# Effects of free play or artificial rules on young soccer players’ individual tactical behaviour: a one-by-one analysis

**DOI:** 10.5114/biolsport.2023.124845

**Published:** 2023-03-06

**Authors:** Asier Gonzalez-Artetxe, Hugo Folgado, José Pino-Ortega, Markel Rico-González, Asier Los Arcos

**Affiliations:** 1 Department of Physical Education and Sport, Faculty of Education and Sport, University of the Basque Country, UPV/EHU, Vitoria-Gasteiz, Basque Country, Spain; 2 Departamento de Desporto e Saúde, Escola de Saúde e Desenvolvimento Humano, Universidade de Évora, Évora, Portugal; 3 Comprehensive Health Research Centre (CRHC), Universidade de Évora, Évora, Portugal; 4 BioVetMed&SportSci Research Group, Faculty of Sports Sciences, University of Murcia, San Javier, Murcia, Spain; 5 Department of Musical, Visual Arts and Physical Education Didactics, Faculty of Education of Bilbao, University of the Basque Country, UPV/EHU, Bilbao, Basque Country, Spain; 6 Society, Sports and Physical Exercise Research Group (GIKAFIT), Department of Physical Education and Sport, Faculty of Education and Sport, University of the Basque Country, UPV/EHU, Vitoria-Gasteiz, Basque Country, Spain

**Keywords:** Training, Learning, Pedagogy, Talent development, Time-motion analysis, Football

## Abstract

This study assessed the effects of playing freely and introducing artificial rules on individual tactical behaviour during the team-possession game phase in two youth soccer categories. Thirty-two developmental players from U-14 and U-16 teams participated in the study, which consisted of four identical training sessions and two test sessions performed before and after the intervention. Each team was divided into two balanced groups, free-play and conditioned, that faced each other during three eight-a-side games (Gk + 7 vs 7 + Gk) in all training sessions. The free-play groups played freely, while the conditioned ones did so constrained by artificial rules. Individual tactical behaviour was assessed during a non-constrained eight-a-side match by the distance to centroid, spatial exploration index, their entropy measures, and the regularity of each player’s displacement on the length and width of the pitch using a local positioning system. In addition to the average outcomes of all the players all together, the one-by-one analysis considered the mean values of each player to appraise individual responses. While the average outcomes of all the players in both groups and categories barely changed (Cohen’s d ≤ small), with a very high inter-player variability, the one-by-one analysis revealed that the training intervention affected each player’s tactical behaviour differently. Introducing artificial rules decreased and raised considerably (Cohen’s d ≥ moderate) in-width and exploratory regularities of most U-14 and U-16 players, respectively. Therefore, assessing the training effects of game-based interventions from the individual to the whole team may provide unique and meaningful insight regarding the tactical competence of each player.

## INTRODUCTION

Ecological dynamics has become a prominent theoretical approach to understanding, from a functional perspective, how and why team-sports players behave and interact in training and performance scenarios [1, 2]. Rooted in ecological dynamics, the nonlinear pedagogy framework [3–5] endorses the design of representative learning environments that simulate competition through the manipulation of task constraints [6–8]. Coaches can implement task constraints to offer players opportunities for action (viz., affordances) that solve the training problem [7, 9]. Task constraints modify the player relation with *teammates* and *adversaries*, *time*, *space*, and *equipment*/*mobile* to conduct players’ emergent tactical behaviour, their spatiotemporal positioning and organization adapting to the specific structural traits of each sport [10], during practice [11, 12].

Recent systematic reviews and meta-analyses [13, 14] reported that task constraints were habitually manipulated in soccer training because of their relevance and easy manageability, those that alter the playing pitch configuration and the number of players involved being the most investigated. Several studies have assessed the acute effects of modifying the playing space on young soccer players’ tactical behaviour and suggested that positioning the goals diagonally [15] or from throw-in line to throw-in line [16], and removing pitch lines (i.e., only corner markings) [17] increased players’ dispersion and impaired synchronization, mostly longitudinal, while adding spatial references such as corridors and sectors helped players to move more regularly but not with greater synchrony [18]. For numerical constraints, it seems that the disequilibrium caused by temporary numerical imbalances [19] or the inclusion of floaters [20] led to players playing wider and slightly prompting their exploratory behaviour. Regarding mobile manipulation, Santos et al. [21] suggested that six-a-side games with a handball and, particularly, rugby balls induced players to explore less and play closer in an unpredictable way. Introducing new artificial rules, coach-made conditions not inherent to the laws of the game are also typical task constraints [6, 13, 14] that may delimit or inhibit specific actions while enhancing or channelling others [7]. For instance, limiting the number of ball touches to one or two during five-a-side games raised players’ synchronization, mainly in-width [22].

Despite the fact that several studies have evaluated the acute effects of wide-ranging task constraints on young soccer players’ tactical behaviour from pitch-positioning-derived variables [13, 14], research about structured training interventions looking for chronic effects or behavioural adaptations to prove the effectiveness of task constraints in applied settings such as soccer academies is still scarce [7]. Santos et al. [23] and Coutinho et al. [24] designed 40 and 20 constraint-based training sessions of approximately 30 min for young Portuguese soccer players and only for attackers, respectively, under the differential learning approach to foster players’ tactical adaptability during small-sided and conditioned games. Both studies compared the chronic effects of traditional training (i.e., control group) with game-based training constrained by the modification of the number of players, playing pitch, number and type of the ball, scoring targets, body restrictions or game rules (i.e., experimental group) and found that central tendency tactical measures barely changed after the differential learning programme, with entropy decrease suggesting a trend in behavioural regularity [23, 24]. More specifically, recent studies assessed the short-term training effects of a single type of task constraint, introducing artificial rules, at different organizational levels [25, 26]. The training intervention applied the nonlinear pedagogy principles of representativeness and constraints manipulation on medium-sided games that faced free-play and conditioned groups of the same team: the first played freely and the second constrained by artificial rules that modified the cooperative motor interactions between teammates. The results indicated that playing under the conditions of artificial rules consolidated collective tactical behaviour during the team-possession game phase [25], while analysing the same intervention at a meso-level [26] revealed that free play could also be valuable to boost team lines’ regularity. Given the behavioural differences at macro- [25] and meso-levels [26], assessing the training effects of free play and artificial rules at the micro-level would be pertinent to provide meaningful insights to coaches for optimizing the training strategies at a more detailed level [2, 27].

In fact, Davids et al. [28] underline that the training strategies need to be designed, selected, and implemented expressly for individual learners to lead each of them to seek an appropriate and individualised functional solution to the problem laid out by the training task, rather than automatizing the same actions for all the players. Introducing or even suppressing (i.e., free play) different task rules for each one of the players during the same game-based task may condition their affordances to modulate their tactical response. For instance, focusing on the team-possession game phase, coaches could manipulate the number of touches, the interaction between players or the use of space by well-known and habitually used artificial rules to compel players to spread out and explore more or to look around for more and better opportunities, guiding their individual tactical behaviour. The individualised analysis makes sense as only a few players in the youth categories become professional, increasing the chances to do so step-by-step [29]. The percentage of European players who signed a contract with a professional club that competed in national leagues increased category-by-category: 3.52% from U-12s, 4.13% from U-14s, 6.63% from U-17s, 16.0% from elite U-19s and between 32.1% and 41.8% of the players belonging to a reserve team of elite Spanish soccer clubs [30–33]. Thus, given that developing tactically competent soccer players requires attending to their individual needs and characteristics, such as playing style, position, or maturity [1, 6–8, 28], and many players will probably not continue in the club in the following years [29–33], assessing the chronic effects of game-based interventions (e.g., free play vs artificial rules) on individual tactical behaviour through one-by-one analysis seems pertinent. The one-by-one analysis considers each player separately to appraise their tactical behaviour and determine individual responses to the same training intervention according, for example, to the playing position.

This study aimed to assess the training effects of modified games playing freely and introducing artificial rules on individual tactical behaviour during the team-possession game phase in two youth soccer categories.

## MATERIALS AND METHODS

### Subjects

Thirty-two Spanish, male youth soccer players from U-14 and U-16 teams of the same club took part in the study. The *a priori* necessary sample size was computed with the G*Power stand-alone power analysis program (version 3.1.9.7 for Windows, Institut für Experimentelle Psychologie, Düsseldorf, Germany) for an effect size of 0.50, an α of 0.05, and a power of 0.80 (1–β) [34]. The total size computed by this method was a minimum of 26 players. The sampling used for the research was non-probability, purposive or convenience sampling and participants were recruited from one of the most representative soccer academies of the region affiliated to a Spanish First Division Club (*LaLiga*). In order not to modify the natural groups for the study, all the players of both teams fully available (i.e., not ill, not injured, nor returning from injury) took part in the intervention (U-14s, *n* = 16; age 13.0 ± 0.40 years; height 1.56 ± 0.07 m; body mass 46.7 ± 5.90 kg; years competing in soccer: 6 ± 1 years; and U-16s, *n* = 16; age 15.9 ± 0.40 years; height 1.70 ± 0.07 m; body mass 57.9 ± 8.30 kg; years competing in soccer: 8 ± 1 years). U-14 and U-16 teams trained twice a week (Tuesdays and Thursdays at 18:00) for ~75 min on an outdoor artificial-turf pitch for 32 and 38 weeks a season, respectively, and competed at weekends in official matches (U-14s for 70 and U-16s for 80 min 11-a-side matches). Both teams competed at the highest competition level for their age, and the U-14s were second in the regular league, so they classified for the Champions League, while the U-16s ended up sixth out of 16 in their league. Participants had soccer identified as their specific sport, trained more than 150 min per week, and competed at the highest local-level competition for their age; they were in Tier 2: Trained/Developmental according to the Participant Classification Framework provided by McKay et al. [35].

The parents, coaches, players, and the club were fully informed about the research procedures, and signed written consent forms for the children’s participation were gathered. All of the participants and their legal guardians were notified about the risks and benefits, and were told that they could withdraw from the study at any time. All of the procedures were carried out in accordance with the Code of Ethics of the World Medical Association (Declaration of Helsinki, 2013) and the standards of the Ethics Committee for Research involving Human Beings of the University of the Basque Country (Code M10_2018_178).

### Study design

Using a controlled study design, each team was divided into two groups according to the coaches’ perception of the players’ technical and tactical level and their position [25, 36], pre-intervention values for the pitch-positioning-derived variables being assessed similarly for both groups (see Results): free-play and conditioned groups (i.e., U-14s-free and U-14s-conditioned; U-16s-free and U-16s-conditioned). Also, playing time in the official matches during the intervention period remained similar for both free-play (70% and 74% of the total match minutes for U-14s-free and U-16s-free, respectively) and conditioned (72% and 69% of the total match minutes for U-14s-conditioned and U-16s-conditioned, respectively) groups. The study took place in the final part of the 2018–2019 season (May) and consisted of four identical training sessions carried out twice a week (Tuesdays and Thursdays) on their habitual training pitch (length = 60 m; width = 40 m) from 18:00 to 19:00, with two test sessions of ~30 minutes (from 18:00 to 18:30) performed before (pre-test) and after (post-test) the training intervention.

The four training sessions that comprised the intervention started with an eight-minute warm-up based on the FIFA 11+ protocol [37] and five minutes of the game tail-tag [38] played by all the players together. After four minutes, the free-play and conditioned groups of each competition category faced each other (i.e., U-14s-free vs U-14s-conditioned and U-16s-free vs U-16s-conditioned) during three seven-minute medium-sided games (Gk + 7 vs 7 + Gk) [39] in all training sessions in the same order, with four minutes of ad libitum rest between them. The free-play group played freely without restrictions and without knowing the playing conditions of the conditioned group, which played under the conditions of artificial rules as key task constraints [3, 6]. Each of the three artificial rules employed in each of the three eight-a-side matches [25] reshaped the relevant structural traits [12, 40] of the modified game setting to attempt to constrain players’ individual tactical behaviour after the training intervention:

1. Relationship with the equipment/mobile: obligation to touch the ball at least three times before passing or shooting at goal could force players to explore more.
2. Relationship with teammates: the player in possession cannot return the ball to the player who had passed to them, which would commit the rest of the teammates to spread out to offer more passing opportunities.
3. Relationship with the space: after passing the ball back (towards their own goal), the next pass had to be a forward pass, which might enhance the regularity of each player’s displacement, especially in-length.

Both free-play and conditioned groups used the same 1-3-3-1 team formation throughout the training intervention with one goalkeeper (Gk), one central defender (CD), two lateral defenders (LD), one central midfielder (CM), two lateral midfielders (LM), and one forward (F), with all the players in their usual playing position. The official game rules, including the offside rule set on the halfway line, were applied during the game. The ball was the standard size (nº 5) and was the same one that was used in official matches by both competition categories. The team coaches presented the training tasks, reminding the free-play group to play freely without restrictions and introducing the artificial rules to the conditioned group. If a player did not respect these rules, then one coach who acted as a referee awarded the opposing team with an indirect free kick from where the offense occurred. Except for this, they remained silent on each lateral line to avoid influencing the participants’ behaviour with their comments or corrections during the game [24, 25]. The coaches provided several balls to avoid losing time and controlled the duration of each task.

The test sessions were carried out on the same pitch and at the same hour before (pre-test) and after (post-test) the intervention. As in training, players had a 48-hour recovery between the previous session or match and the test sessions and committed to continuing with their daily activities, abstaining from doing uncommon and too intense exercises during the intervention period. To control the stimulus of the players by a controlled activity, both test sessions started with the FIFA 11+ warm-up programme [37] and a 30-meter sprint. Then, the individual tactical behaviour was assessed during a non-constrained seven-minute eight-a-side match, in which the free-play and conditioned groups of each competition category faced each other. The game rules, team formation and playing positions, ball replacement, and the coach intervention conditions were the same as those used in the training intervention during the pre-test and the post-test. Similarly as in the intervention, two researchers (AGA and ALA) were present pitch-side from the start to the end of the training and test sessions, and noted all of their observations in a field diary to guarantee the fidelity of the research procedure.

### Data collection

Positional data were gathered to assess individual tactical behaviour by a time-motion tracking system using a local positioning system (LPS) (IMU; WIMU PRO, RealTrack Systems, Almeria, Spain) based on ultra-wideband (UWB) technology. The UWB system consists of a reference system and tracking devices that were worn in a special vest by all of the players, who had been previously familiarized with the use of these devices during several training sessions. The reference system is composed of antennae that are transmitters and receivers of the radio-frequency signals [41]. The antennae (mainly the master antenna) computerize the position of the devices that are in the playing area, while the device receives that calculation using time difference of arrival (TDOA) [42, 43]. These lightweight (70 g) inertial devices of 81 × 45 × 16 mm with 2 GB flash memory and five-hour battery life incorporate both GPS (10 Hz) and UWB (18 Hz) sensors, together with triaxial accelerometers (four sensors < 1000 Hz), triaxial gyroscopes (three sensors < 1000 Hz), and triaxial magnetometers (160 Hz) [41, 42]. Six antennae forming a hexagon to better signal emission and reception at a height of three meters and held by a tripod were set up on a training pitch away from metallic materials, with similar environmental conditions on both days (cool temperatures: 15.9°C and 17.3°C; humidity gradients: 60% and 51%; and slow air circulation: 12.3 ^km^/_h_ and 11.8 ^km^/_h_ for pre-test and post-test sessions, respectively) that allow easier positioning [41, 42]. The validity and inter-unit reliability of this equipment for tactical analysis had been confirmed during a continuous test composed of five courses (i.e., perimeter of the pitch course, half-way line, centre circle, perimeter of the penalty area, and semi-circular penalty area) and a small-sided game that emulated real conditions [41], with a mean absolute error of less than 10 cm (*x* coordinate: 9.57 ± 2.66 cm; *y* coordinate: 7.15 ± 2.62 cm) and a typical error of measurement of 1–1.15% for surface area. Furthermore, WIMU PRO had been certified the International Match Standard (IMS) from FIFA and, according to a survey to assess the quality of the data [43] to measure the players’ tactical behaviour, data collection was satisfactory (90% score).

The raw data were sampled at 18 Hz and then interpolated to obtain 100 data points per second for better computational time management and easier comparison with existing studies with a similar methodology [26]. Additionally, testing of eight-a-side matches was recorded at 240 frames per second (fps) at FullHD quality (1920 × 1080 pixels) with two iPhone 7 Plus devices (Apple Inc., Cupertino, Ca, USA) that were located four meters above the playing pitch to differentiate the team-possession and non-possession game phases. The data were downloaded and synchronized with the video using S PRO software (RealTrack Systems, Almeria, Spain) and processed using MATLAB (MATLAB [2018a], The MathWorks Inc., Natick, Ma, USA) following the existing procedures [44].

### Pitch-positioning-derived variables

All of the measurements were carried out for the team-possession game phase. The criteria established by Castellano [45] were applied to differentiate ball possession for each team and exclude stoppages in play. Given the difference in length of the several ball possessions, only those that lasted more than five seconds (i.e., 500 data points) were considered for further analysis [46]. Intra- and inter-observer agreement was evaluated using Cohen’s kappa statistic to ensure the reliability of data collection and for differentiation of team-possession and non-possession game phases. The same randomly selected match was observed twice by AGA and once by ALA using the LINCE PLUS Research Software for Behaviour Video Analysis [47]. The corresponding calculations were performed in the free software GSEQ5.1 [48, 49]. Satisfactory intra- and inter-observer agreement was obtained for the alignment of ball possessions (intra-observer: 0.93 ± 2 events tolerance, inter-observer: 0.90 ± 2 events tolerance) and the duration of the team-possession game phase (intra-observer: 0.96 ± 2 seconds tolerance, inter-observer: 0.94 ± 2 seconds tolerance) [50].

Individual tactical behaviour was assessed by: 1. the distance of each outfield player to the team centroid (DC – Formula 1); 2. spatial exploration index (SEI – Formula 2) – the distance of each outfield player to their own mean position throughout the game; and 3. the regularity of each outfield player displacement on the length and width of the pitch [27, 51]. To better describe the stochastic and complex nature of the soccer game [27, 51], the outcomes were processed using both central tendency and approximate entropy normalized (ApEn *norm*) measures [46], which were calculated by MATLAB with a vector length (m) of two and a tolerance (r) of 0.2 × *SD* of the time series to assess the predictability of the individual tactical behaviour (Formula 3). Higher entropy values reflect more unpredictability, while lower values manifest more regularity.

$$DC_{k\left(i\right)}=\sqrt{{\left(P_{xk\left(i\right)}-C_{x\left(i\right)}\right)}^{2}+{\left(P_{yk\left(i\right)}-C_{y\left(i\right)}\right)}^{2}}$$

Formula 1 – Distance to centroid (DC) calculation, where *C* represents the centroid or geometrical centre of the team at any given instant *i*, *k* the individual player for whom the DC is being calculated, *Px* and *Py* the players’ position coordinates for the *×* and *y* axis, respectively, at instant *i*.

$$SEI_{k\left(i\right)}=\sqrt{{\left(P_{xk\left(i\right)}-P_{xk\left(m\right)}\right)}^{2}+{\left(P_{yk\left(i\right)}-P_{yk\left(m\right)}\right)}^{2}}$$

Formula 2 – Spatial exploration index (SEI) calculation, where *Px* and *Py* represent the players’ position coordinates for the *×* and *y* axis, respectively, at instant *i* or as a mean (*m*) position, and *k* the individual player for whom the SEI is being calculated.

$$ApEn_{norm}=\frac{ApEn{\left(2,0.2,N\right)}_{TS}}{\sum_{i}^{100}ApEn{\left(2,0.2,N\right)}_{Ui}/100}$$

Formula 3 – Normalized approximate entropy (ApEn *norm*) calculation, where the regularity of the time series TS is normalized using the ratio between the original ApEn and the mean ApEn calculated in 100 random series *U*_i_.

### Statistical analysis

Descriptive outcomes of each variable (means ± standard deviations [*SD*]) were computed using the mean value for each ball possession with more than 500 data points (i.e., five seconds). Intra-group and intra-player practical differences were assessed by Cohen’s *d* effect size and percentage change from the pre-test to the post-test. The thresholds for effect size (*d*) were 0.25, trivial; 0.50, small; 1.0, moderate; and > 1.0, large [52]. The coefficient of variation (CV % = (SD/mean) × 100) for average outcomes was also calculated to assess inter-player variability. Apart from the average outcomes of all of the players all together, the one-by-one analysis considered the mean value of each player for each ball possession of the team-possession game phase to assess and compare their individual tactical response (means ± *SD*) from the pre-test to the post-test. Statistical procedures were performed with jamovi computer software (The jamovi project [2022], version 2.3.2).

## RESULTS

No considerable differences (Cohen’s *d* ≤ small) between intervention groups of U-14s (DC *d* = 0.26; DC _ApEn norm_ *d* = 0.10; SEI *d* = 0.37; SEI _ApEn norm_ *d* = 0.10; length regularity [ApEn *norm*] *d* = 0.13; and width regularity [ApEn *norm*] *d* = 0.13) and U-16s (DC *d* = 0.18; SEI *d* = 0.19; SEI _ApEn norm_ *d* = 0.20; length regularity [ApEn *norm*] *d* = 0.14; and width regularity [ApEn *norm*] *d* = 0.25) were apparent in the baseline comparison, except for the DC _ApEn norm_ (*d* = 0.57) of U-16s players.

In general, the average outcomes of all outfield players of each free-play and conditioned groups for both competition categories (i.e., U-14s and U-16s) barely changed (*d* ≤ small) after the training intervention (Table 1). Inter-player variability was considerable, from 35.1% to 200%, for all the measurements (Table 1).

**TABLE 1:** Average outcomes for U-14s’ and U-16s’ individual tactical behaviour. Descriptive (mean ± *SD*; CV) and intra-group comparison (% change and Cohen’s *d*) for both free-play and conditioned groups.

Free-play group	Pre-test		Post-test		Change	Cohen’s *d*	
	mean ± SD	CV (%)	mean ± SD	CV (%)	(%)	*d*	Int.
*U-14s*							
DC (m)	10.4 ± 4.75	45.7	10.9 ± 3.87	35.5	4.80	0.12	Trivial
DC _ApEn norm_	0.28 ± 0.19	67.9	0.22 ± 0.15	68.2	-21.4	0.35	Small
SEI(m)	9.81 ± 3.86	39.3	10.2 ± 3.24	31.8	3.98	0.11	Trivial
SEI _ApEn norm_	0.07 ± 0.11	157	0.07 ± 0.10	143	0.00	0.00	Trivial
Length _ApEn norm_	0.06 ± 0.07	117	0.07 ± 0.12	171	16.7	0.10	Trivial
Width _ApEn norm_	0.06 ± 0.07	117	0.08 ± 0.12	150	-33.3	0.20	Trivial
*U-16s*							
DC (m)	12.5 ± 4.39	35.1	11.2 ± 4.49	40.1	-10.4	0.29	Small
DC _ApEn norm_	0.21 ± 0.16	76.2	0.31 ± 0.22	71.0	47.6	0.52	Moderate
SEI(m)	9.07 ± 3.40	37.5	10.5 ± 4.30	41.0	15.8	0.37	Small
SEI _ApEn norm_	0.06 ± 0.08	133	0.05 ± 0.08	160	-16.7	0.12	Trivial
Length _ApEn norm_	0.04 ± 0.06	150	0.05 ± 0.07	140	25.00	0.15	Trivial
Width _ApEn norm_	0.07 ± 0.10	143	0.07 ± 0.12	171	0.00	0.00	Trivial

**Table:** 

Conditioned group	Pre-test		Post-test		Change	Cohen’s *d*	
	mean ± SD	CV (%)	mean ± SD	CV (%)	(%)	*d*	Int.
*U-14s*							
DC (m)	11.6 ± 4.61	39.7	12.1 ± 4.65	38.4	4.31	0.11	Trivial
DC _ApEn norm_	0.30 ± 0.22	73.3	0.25 ± 0.22	88.0	-16.7	0.23	Trivial
SEI(m)	8.37 ± 3.94	47.1	8.60 ± 4.41	51.3	2.75	0.06	Trivial
SEI _ApEn norm_	0.06 ± 0.08	133	0.05 ± 0.07	140	-16.7	0.13	Trivial
Length _ApEn norm_	0.07 ± 0.08	114	0.06 ± 0.12	200	-14.3	0.10	Trivial
Width _ApEn norm_	0.07 ± 0.10	143	0.05 ± 0.06	120	-28.6	0.24	Trivial
*U-16s*							
DC (m)	11.6 ± 5.76	49.7	11.9 ± 5.30	44.5	2.59	0.05	Trivial
DC _ApEn norm_	0.32 ± 0.22	68.8	0.28 ± 0.16	57.1	-12.5	0.21	Trivial
SEI(m)	9.85 ± 4.65	47.2	8.82 ± 4.61	52.3	-10.5	0.22	Trivial
SEI _ApEn norm_	0.08 ± 0.12	150	0.04 ± 0.06	150	-50.0	0.42	Small
Length _ApEn norm_	0.05 ± 0.08	160	0.03 ± 0.04	133	-40.0	0.32	Small
Width _ApEn norm_	0.05 ± 0.05	100	0.05 ± 0.08	160	0.00	0.00	Trivial

DC = distance to centroid; SEI = spatial exploration index; ApEn *norm* = normalized approximate entropy (arbitrary units); SD = standard deviation; CV = coefficient of variation; Change = percentage change; *d* = Cohen effect size; Int. = effect size’ qualitative interpretation.

The one-by-one analysis indicated that the training intervention affected each player’s tactical response differently. Altogether, the U-14s’ DC and SEI central tendency and ApEn *norm* measures (Figures 1a and 2a) barely changed (d ≤ small) for both free-play and conditioned groups. The U-14s’ displacement regularity on the width of the pitch (Figure 3a) decreased considerably (d ≥ moderate) for most of the outfield players (^4^/_7_) who played conditioned by artificial rules during the training intervention.

**FIG. 1a:**
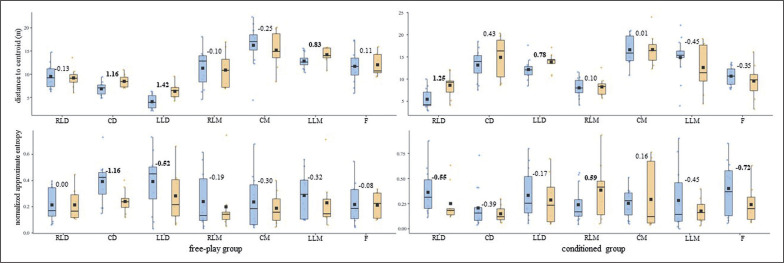
U-14s players’ distance to centroid central tendency and normalized approximate entropy (arbitrary units) measures for both free-play and conditioned groups. Intra-player comparison (Cohen’s *d*) between pre-test (blue) and post-test (orange) boxes, moderate – large effect sizes in bold. RLD = right lateral defender; CD = central defender; LLD = left lateral defender; RLM = right lateral midfielder; CM = central midfielder; LLM = left lateral midfielder; F = forward.

**FIG. 1b:**
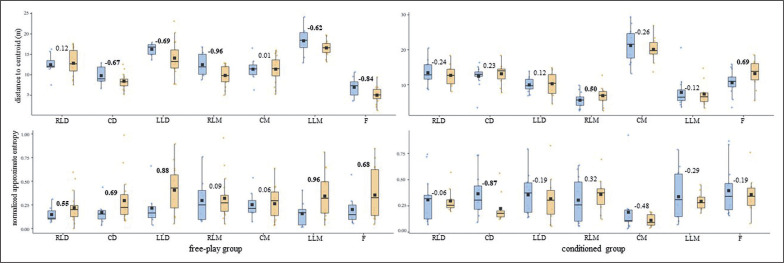
U-16s players’ distance to centroid central tendency and normalized approximate entropy (arbitrary units) measures for both free-play and conditioned groups. Intra-player comparison (Cohen’s *d*) between pre-test (blue) and post-test (orange) boxes, moderate – large effect sizes in bold. RLD = right lateral defender; CD = central defender; LLD = left lateral defender; RLM = right lateral midfielder; CM = central midfielder; LLM = left lateral midfielder; F = forward.

**FIG. 2a:**
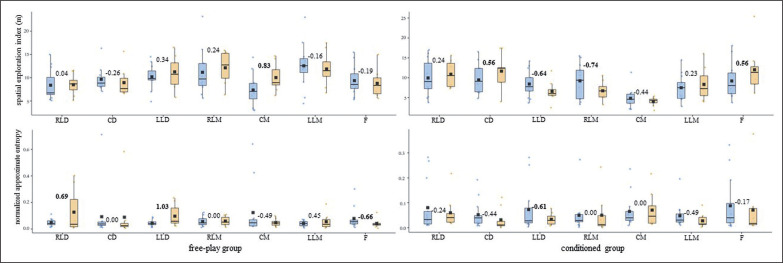
U-14s players’ spatial exploration index central tendency and normalized approximate entropy (arbitrary units) measures for both free-play and conditioned groups. Intra-player comparison (Cohen’s *d*) between pre-test (blue) and post-test (orange) boxes, moderate – large effect sizes in bold. RLD = right lateral defender; CD = central defender; LLD = left lateral defender; RLM = right lateral midfielder; CM = central midfielder; LLM = left lateral midfielder; F = forward.

**FIG. 2b:**
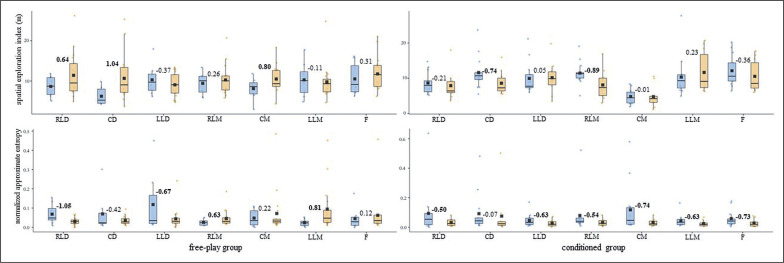
U-16s players’ spatial exploration index central tendency and normalized approximate entropy (arbitrary units) measures for both free-play and conditioned groups. Intra-player comparison (Cohen’s *d*) between pre-test (blue) and post-test (orange) boxes, moderate – large effect sizes in bold. RLD = right lateral defender; CD = central defender; LLD = left lateral defender; RLM = right lateral midfielder; CM = central midfielder; LLM = left lateral midfielder; F = forward.

**FIG. 3a:**
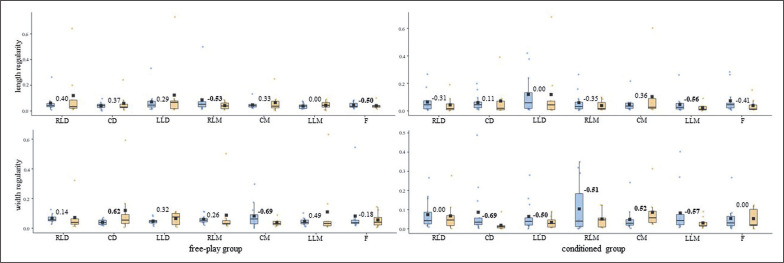
U-14s players’ displacement regularity (normalized approximate entropy measures [arbitrary units]) on the length and width of the field for both free-play and conditioned groups. Intra-player comparison (Cohen’s *d*) between pre-test (blue) and post-test (orange) boxes, moderate – large effect sizes in bold. RLD = right lateral defender; CD = central defender; LLD = left lateral defender; RLM = right lateral midfielder; CM = central midfielder; LLM = left lateral midfielder; F = forward.

**FIG. 3b:**
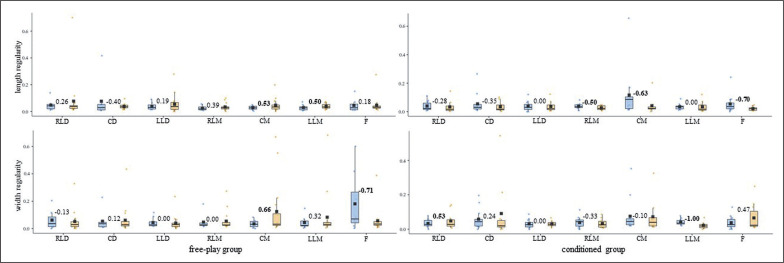
U-16s players’ displacement regularity (normalized approximate entropy measures [arbitrary units]) on the length and width of the field for both free-play and conditioned groups. Intra-player comparison (Cohen’s *d*) between pre-test (blue) and post-test (orange) boxes, moderate – large effect sizes in bold. RLD = right lateral defender; CD = central defender; LLD = left lateral defender; RLM = right lateral midfielder; CM = central midfielder; LLM = left lateral midfielder; F = forward.

While most of the U-16s-free players’ (^5^/_7_) DC central tendency measures diminished considerably (*d* ≥ moderate), their ApEn *norm* rose (^5^/_7_, *d* ≥ moderate) (Figure 1b). The U-16s-conditioned players’ (^6^/_7_) SEI tended considerably (*d* ≥ moderate) toward regularity after the training intervention (Figure 2b). The U-16s’ displacement regularity on the length and width of the pitch (Figure 3b) barely changed (d ≤ small) for both free-play and conditioned groups from the pre-test to the post-test.

## DISCUSSION

To the best of our knowledge, this is the first study to assess the training effects on individual tactical behaviour from pitch-positioning-derived variables through one-by-one analysis. While the average outcomes of all of the outfield players of each group (i.e., free-play and conditioned groups) for both competition categories (i.e., U-14s and U-16s) all together barely changed (*d* ≤ small) (Table 1), the one-by-one analysis revealed that the training intervention affected each player’s tactical behaviour differently (Figures 1, 2, and 3). Attending to these outcomes, together with previous studies that had analysed tactical behaviour at macro- [25] and meso-levels [26], it seems necessary to delve into the different organizational levels of soccer teams to endeavour to comprehend the complex, social, and adaptive nature of the game [2, 27].

The average outcomes of the distinct individual metrics (i.e., DC and SEI central tendency and ApEn *norm* measures and displacement regularity on the length and width of the pitch) that were selected to assess individual tactical behaviour in a non-constrained testing match barely changed (*d* ≤ small) after the short-term training intervention. Similarly, Coutinho et al. [24] found that the average outcomes of DC and SEI central tendency measures and displacement regularity barely changed (*d* ≤ small) after a lengthier (i.e., 10 weeks) constraint-based training programme only for young (i.e., U-15s and U-17s) attackers. At different organizational levels, the previous studies suggest that playing freely or conditioned by artificial rules could impact considerably (*d* ≥ moderate) on the collective tactical variables (i.e., the change in the centroid position, the interpersonal distance between teammates, and surface area central tendency and entropy measures) that are utilized to assess the team [25] and team lines’ [26] tactical behaviour. In addition to the different metrics employed to assess tactical behaviour at macro-, meso-, and micro-levels [53], distinct complexity principles such as the emergence phenomenon, self-organization, and adaptation could account for these differences [54, 55]: team and team lines’ behaviours emerge from the nonlinear dynamic interactions between players in an unpredictable way and without a blueprint. The players may self-organize to display collective structures and behaviours at larger scales, adapting through learning and cooperation with teammates to reconfigure their tactical behaviour at multiple levels [54, 55]. Therefore, unlike collective tactical behaviour [25, 26], it seems that different game-based interventions that jointly combine several task constraints did not considerably influence players’ average individual tactical behaviour (Table 1) [24].

In light of the average outcomes of all of the players and the very high inter-player variability, primarily for entropy measures, that were obtained by this (Table 1) and prior studies [24–26], the one-by-one analysis seems to be essential to deepen the effects of different training strategies on young soccer players’ individual tactical behaviour. While the majority of U-14s’ DC and SEI central tendency and ApEn *norm* measures (Figures 1a and 2a) barely changed (d ≤ small) after the training intervention, their displacement regularity on the width of the pitch decreased considerably (d ≥ moderate) for most of the conditioned group players (^4^/_7_). This is in line with a team tactical behaviour study [25], in which the collective tactical behaviour tended toward regularity after introducing artificial rules as key task constraints. Meanwhile, even though the majority of the U-16s’ displacement regularity (Figure 3b) barely changed (d ≤ small), DC (Figure 1b) and SEI (Figure 2b) one-by-one outcomes considerably changed (*d* ≥ moderate) after the training intervention. It is worth mentioning here that the DC central tendency measures decreased, and their ApEn *norm* increased considerably (*d* ≥ moderate) for most of the players (^5^/_7_ in both cases) who played freely during the four training sessions. Hence, future research could ask whether getting closer to the team centroid raised the players’ unpredictability. Regarding the SEI, similarly to the entropy measures of the use of space (i.e., surface area) at a macro-level [25], the ApEn *norm* dropped for six of the seven U-16s-conditioned players. This suggests that introducing artificial rules could boost the exploratory regularity.

Since the adaptations in young soccer players’ tactical behaviour depends on the use of three artificial rules during the intervention, changes cannot be attributed to a determinate task condition. So, a broad interpretation of the training effects is required. For practical purposes, just like in lengthier structured constraint-based programmes [24], coaches should not expect considerable behavioural changes in their players’ average tactical response after a short-term intervention, with very high inter-player variability. Even so, free play or the combination of three artificial rules impacted each player’s tactical behaviour in a different way, mainly those who played conditioned during the training intervention. Thus, generalizing the effects of game-based interventions to the whole squad would not be suitable [7, 8, 28]. As such, academy heads and youth soccer coaches could take advantage of the one-by-one analysis to supervise the individual’s development, attend to their particular needs and characteristics, and adapt and optimize their training strategies.

## CONCLUSIONS

This study is the first to assess training effects on the individual tactical behaviour from pitch-positioning-derived variables through one-by-one analysis. This analysis seems to be crucial to study the different organizational levels of soccer teams in depth and comprehend the complex, social, and adaptive nature of the game. Given that the average outcomes of all of the outfield players of both free-play and conditioned groups for both U-14 and U-16 competition categories together barely changed (*d* ≤ small), an individualized analysis could be valuable to complement the players’ average measures. Indeed, the one-by-one analysis revealed that the training intervention affected each player’s tactical behaviour differently. Therefore, assessing the training effects of game-based interventions from the individual to the whole team may provide unique and meaningful insight regarding the tactical competence of each player.
